# Potential function exploration of lncRNAs in idiopathic pulmonary fibrosis: insights from whole transcriptome sequencing data analysis

**DOI:** 10.1016/j.clinsp.2025.100732

**Published:** 2025-08-08

**Authors:** Xinran Ma, Dong Chen, Chenghai Li, Wenjuan Wu

**Affiliations:** a People's Hospital of Zhengzhou University, Zhengzhou, China; b Wuhan Ruixing Biotechnology Co. Ltd., Wuhan, China; c Stem Cell Program at Henan Provincial People's Hospital and People's Hospital of Zhengzhou University, China; d Department of Geriatric Medicine, Henan Provincial People's Hospital and Provincial Hospital Clinical Medical College of Zhengzhou University, Zhengzhou, China

**Keywords:** Idiopathic pulmonary fibrosis, RNA-seq, LncRNAs, Co-expression, Inflammatory response

## Abstract

**Background:**

Research has shown that long noncoding RNAs (lncRNAs) play a role in Idiopathic Pulmonary Fibrosis (IPF), but their specific functions and patterns of expression are still unclear.

**Method:**

A diagnostic study was conducted by utilizing analysis techniques. RNA sequencing (RNA-seq) data from 12 IPF patients and 5 controls was used to study lncRNA functions in IPF. The authors identified Differentially Expressed lncRNAs (DElncRNAs) and explored co-expression networks in a transient manner, as well as using Weighted Gene Co-expression Network Analysis (WGCNA) to identify modules associated with IPF.

**Results:**

The study found 541 differentially expressed lncRNAs in IPF, with 201 up-regulated and 340 down-regulated. DElncRNAs, especially the up-regulated, were significantly correlated with DEmRNAs at their expression levels. DEmRNAs showed extracellular matrix-related biological functions in addition to increased lncRNAs. WGCNA results demonstrated that Module Eigengene green (MEgreen) and MEred modules indicated the highest negative correlation significance with IPF phenotype, and eigengene patterns of both modules were downregulated in IPF samples. The authors identified six significant lncRNAs in these two modules, including FAM13A-AS1, RP11–180C16.1, MYO16-AS1, AC007278.2, BACH1-IT2, and RP11–153M7.5, and their co-expressed DE mRNAs were enriched in inflammatory response pathways. The authors used single-cell RNA sequencing (scRNA-seq) data to investigate dysregulated lncRNAs and their co-expressed mRNAs. The authors found that five DEmRNAs that were co-expressed with DElncRNAs exhibited dysregulated expression patterns in multiple cell types of the IPF samples.

**Conclusion:**

LncRNAs are functionally active and potentially involved in the inflammatory response in pathological processes of IPF. It is also important to consider some specific lncRNAs as potential diagnostic biomarkers or therapeutic targets for preclinical and clinical studies with IPF in the future.

## Highlights

• Long Noncoding RNAs (lncRNAs) played a role in Idiopathic Pulmonary Fibrosis (IPF).
• Their specific functions and patterns of expression are still unclear.
• The authors conducted a diagnostic study to study lncRNA functions in IPF.
• lncRNAs were functionally active and potentially involved in inflammatory response.
• Specific lncRNAs might be promising diagnostic biomarkers or therapeutic targets.

## Introduction

Idiopathic Pulmonary Fibrosis (IPF) is a chronic lung disease characterized by progressive scarring and causes epithelial cells to die, fibroblasts to proliferate, excess Extracellular Matrix (ECM) to accumulate, the destruction of pulmonary vessels, and loss of alveolar gas exchange units.^1^^,^^2^ IPF is the most common disease in elderly individuals, with a prevalence of 494 cases per 100,000 in adults over 65 in 2011, which was twice as much as that in the prevalence recorded 10-years earlier.^3^ Patients with IPF are affected both physically and emotionally, and patient numbers are rising in recent years.^4^^,^^5^ Although many pathobiological concepts are emerging, including senescence, oxidative stress and cellular plasticity, the exact ways in which IPF works are not completely known.^6^

Long Noncoding RNA (lncRNA) is a diverse type of RNA over 200 nucleotides in length and does not code for proteins. LncRNAs do not code for proteins but can interact with different cellular molecules, including mRNAs, to control cell fate.^7^^,^^8^ LncRNAs play a role in the development of different diseases, including tumors, infection, and other extensive diseases, through regulating gene expression at different stages, including transcription and post-transcription.^7^ Interestingly, lncRNAs are supposed to be critical regulators and prognostic markers in IPF. For example, Fibrosis is induced by Lnc949 mediated by FKBP5.^9^ Targeting lncRNA H19 can enhance IPF treatment by acting as a sponge for miR-196a.^10^ LncRNA ZEB1-AS1 promotes pulmonary fibrosis by inducing epithelial-mesenchymal transition through ZEB1.^11^ Age-related IPF is predicted by upregulation of lncRNA AP003419.16.^12^ By targeting miR-138, PFAR promotes lung fibrogenesis and activation of the YAP1-Twist axis.^13^ IPF progression is prevented by LncRNA CTD-2528L19.6 by alleviating fibroblast activation.^14^ LncRNA Hoxaas3 promotes lung fibrosis via miR-450b-5p-mediated regulation of Runx1.^15^ Alveolar epithelial cells are induced to undergo EMT by ChRF through inhibiting miR-146a and upregulating L1CAM.^16^ IPF is suppressed when microRNA-369–3p/TRIM2 is knocked down by DLEU2 knockdown.^17^ CTGF promotes lung fibrosis by competing with lncRNA PFAL for miR-18a.^18^ Inhibiting lung fibroblast proliferation through reduced levels of β-Catenin is achieved by lncRNA FENDRR.^19^ As mentioned above, the main process has been observed to understand the basic mechanisms of IPF and find targets for treatment. Therefore, lncRNAs have drawn attention for their roles in IPF and the authors believe that there are still a large number of unidentified lncRNAs to be implicated in the progression of IPF.

In this study, the authors suggest that certain unidentified lncRNAs may play a role in the development of IPF. The authors analyzed high-throughput RNA-seq and single-cell RNA-seq (scRNA-seq) datasets from the GEO database, including GSE138283^20^ and GSE122960,^21^ respectively, to compare lung tissue samples from IPF patients and healthy controls. Using the ribosome elimination method to extract RNAs, the authors constructed a transcriptome library and identified lncRNAs that may be involved in inflammation by interacting with differentially expressed mRNAs. This study is one of the first studies linking several novel lncRNAs to IPF inflammatory pathways using both bulk and single-cell transcriptomics and may provide new insights into potential therapeutic targets for DE lncRNAs in IPF in future studies.

## Materials and methods

### Obtaining and processing of the public transcriptome sequencing data

By using the NCBI SRA Tool fastq-dump and FASTX-Toolkit, Run files of the public dataset GSE138283 from the Sequence Read Archive (SRA) were downloaded and converted to fastq format after pruning low-quality bases (v.0.0.13; http://hannonlab.cshl.edu/fastx_toolkit/). FastQC was performed for a clean read evaluation (http://www.bioinformatics.babraham.ac.uk/projects/fastqc). Due to the absence of metadata, clinical variations were not controlled in this study. This study adheres to the relevant guidelines for reporting research. The authors followed the STROBE Statement to ensure the rigor and transparency of the present study design, analysis, and reporting.

Meanwhile, a single cell RNA-seq (scRNA-seq) dataset PRJNA507000^21^ of lung transplantation samples from three elderly controls and three young controls, as well as four IPF patients, was determined by Unique Molecular Identifier (UMI) (https://www.ncbi.nlm.nih.gov/bioproject/PRJNA507000). The UMI count matrix was transformed into a Seurat matrix with the Seurat R package (version 4.0.4),^22^ Low-quality cells were excluded from the study if their UMI count exceeded 1000, or their detectable gene count exceeded 500, or the mitochondrial-derived UMI count exceeded 15 %.

### Data pre-processing and quality control for the scRNA-seq analysis

To generate possible anchors, the top 2000 variable genes were utilized in Seurat's Find Integration Anchors feature, followed by data integration using the Integrate Data function. The authors used Seurat's Elbow plot function to reduce the dimensionality of scRNA-Seq datasets by performing PCA on them. In Seurat's Find Clusters function, the default resolution was set in order to identify the main clusters of cells (res = 0.4). Using tSNE or UMAP plots, the authors visualized 25 major cell types. In the Seurat package, the “Find Markers” function was used to identify genes for each cluster of cells (v4.0.4), and the ScType tool^23^ was used to annotate previously published pulmonary fibrosis markers.^24^

### Analyzing differentially expressed genes (DEGs) based on read alignment

The alignment of clean data to GRCh38 was permitted with four mismatches.^25^ Gene read numbers and FPKM were calculated based on uniquely mapped reads. Gene expression levels were assessed by using FPKM. In order to identify DEGs, the authors detected RNA-seq data using DEseq2,^26^ a tool specifically developed for analyzing gene expression levels.

The authors assessed gene expression differences by examining fold change (FC ≥ 2 or ≤ 0.5) and false discovery rate (FDR ≤0.05) to identify genes that were differently expressed.

### Predictions and identifications of lncRNA directions

The present analysis of lncRNA expression patterns was driven by Cufflinks software, which utilizes a pipeline for identifying lncRNAs similar to previous studies.

### Analysis of co-expression networks and weighted gene co-expression networks (WGCNA)

Gene expression patterns were recognized by the WGCNA package (version 1.73)[27] by clustering genes with mimicking expression patterns with default parameters. Module preservation statistics and sensitivity analyses were run. Clustered modules are represented by eigengenes, which are genes whose expression patterns are similar. Automatic network construction with an optimal soft thresholding power of β = 8 and a minimum of 30 genes in one module was used. According to Pearson's Correlation Coefficients (PCCs), the lncRNAs and their host mRNAs were classified into three classes: correlations, both positively and negatively, as well as non-correlations.

### Analyzing functional enrichment

By using GO terms and KEGG pathways from the KOBAS 2.0 server, the authors performed a systematic functional classification of DEGs.^28^ Using Hypergeometric Testing and Benjamini-Hochberg FDR controls, each term was enriched. Functional enrichment was analyzed using Reactome's (http://reactome.org) pathway profiling.

### Other statistical analysis

The authors used factoextra in *R* to visualize sample clustering with PCA's first two components (https://cloud.r-project.org/package=facroextra). The *R* script heatmap visualized normalized NGS data and genomic annotations(https://cran.r-project.org/web/packages/pheatmap/index.html). By using the Student’s *t*-test, two groups were compared.

## Results

### Genome-wide profiling of the lncRNA expression in IPF

**Fig. 1:**
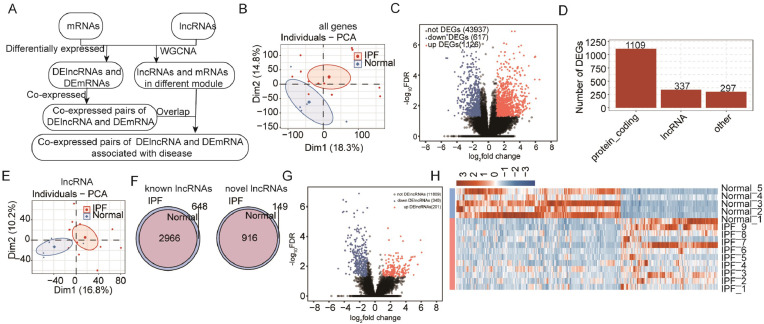
Abundant lncRNAs detected in Idiopathic Pulmonary Fibrosis (IPF) samples and the control samples. (A) The flow chart showed the construction process of the mRNA and lncRNA network. (B) Principal Component Analysis (PCA) of 12 IPF and 5 normal samples based on normalized expression levels of all known genes. (C) Volcano plot presented Differentially Expressed Genes (DEGs) in IPF samples compared with normal samples in each group as experimental replicates. Red indicates up-regulated genes (FC ≥ 2 and FDR ≤ 0.05) and blue indicates down-regulated genes (FC ≤ 0.5 and FDR ≤ 0.05). (D) The bar chart showed the number of different types of genes (including protein-coding genes, annotated lncRNAs, and others). (E) Principal Component Analysis (PCA) of 12 IPF and 5 normal samples based on normalized lncRNAs (including predicted novel lncRNAs) expression levels. (F) Venn diagram of detected annotated lncRNAs (left) and predicted novel lncRNAs (right) in IPF and normal samples. At least two samples with FPKM ≥ 0.2 were considered to be detected in the group. (G) Volcano plot presented differentially expressed lncRNAs in IPF compared with normal samples in each group as experimental replicates. Red indicates up-regulated genes (FC ≥ 2 and FDR ≤ 0.05) and blue indicates down-regulated genes (FC ≤ 0.5 and FDR ≤ 0.05). H. Expression heatmap of all significant DE lncRNAs in IPF and normal samples.

To study lncRNA expression patterns and functions in IPF, a systematic analysis of RNA-seq dataset (GSE138283) between the IPF and the controls^20^ was performed in this study. The lung biopsy samples were obtained from 12 IPF patients and 5 controls. By depleting ribosomal RNAs from biopsy samples, the RNA-seq dataset was prepared to analyze lncRNA expression and functions. After acquiring high-quality data and aligning them to the human genome, the lncRNA prediction procedure and following differential expression and co-expression analysis between mRNAs and lncRNAs were analyzed according to the methods previously published.^29^ The overall design of this manuscript was presented in Fig. 1A. The Pearson correlation coefficients among different samples were calculated and presented in the diagonal heatmap (Supplemental Fig. 1A). It was shown that two control samples were distributed among IPF samples and the other three control samples were clustered together, indicating the individual variation of tissue samples. PCA was used to analyze gene expression levels in both the IPF and control groups to study sample clustering. The confidence ellipses of the two groups showed no overlap (Fig. 1B). With the criteria of |log_2_ fold change| > 1 and False Discovery Rate (FDR) < 0.05, 1743 genes were differentially expressed in the IPF group, including 1126 up-regulated and 617 down-regulated genes (Fig. 1C). Among 1743 DEGs, 1109 were protein-coding genes; 337 were lncRNAs and 297 were other types (Fig. 1D). The protein-coding genes and lncRNAs account for 83.0 % of the total DEGs, indicating their important roles in IPF. The functions of the DE mRNAs were analyzed using GO enrichment analysis. The up-regulated mRNAs were mainly enriched in extracellular matrix organization and tumor necrosis factor-mediated signaling pathways. The decreased mRNAs were primarily involved in immune response to fungus, inflammation regulation, and cell signaling pathways (Supplemental Fig. 1B‒C). These studies showed that extracellular matrix and inflammation-related signal pathways play important roles in the pathogenesis of IPF. In the following part of this research, the authors will focus on the expression patterns and functions of lncRNAs.

Further exploration of lncRNAs in IPF revealed separation of detected lncRNAs in both IPF and control groups based on PCA results (Fig. 1E). As known, 4679 lncRNAs were detected in total, including 3614 known and 1065 novel lncRNAs. All known lncRNAs in the IPF group were included in the control group, and 648 novel lncRNAs were specific to the control group. A similar result was observed for novel lncRNAs, with 916 common lncRNAs in both groups, and 149 specific lncRNAs in the control group (Fig. 1F). In IPF, 541 common lncRNAs showed differential expression, with 201 up-regulated and 340 down-regulated (Fig. 1G). The detailed number of the DE known and novel lncRNAs can be acquired in Supplemental Figure 1D The DE lncRNAs heatmap displayed strong consistency across the samples (Fig. 1H). The results indicate that lncRNAs are actively expressed and differ between IPF and control samples, suggesting their potential importance in IPF.

### DE lncRNAs correlated with DE mRNAs in a trans manner

**Fig. 2:**
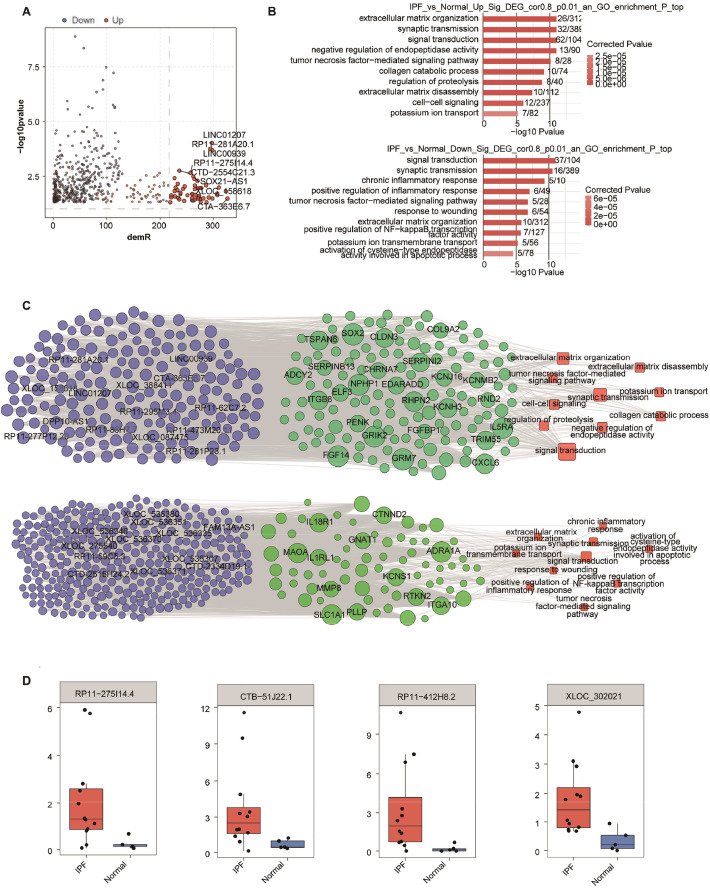
Co-expression network illustration between DE lncRNAs and DE mRNAs. (A) Scatter plot showed DE lncRNAs in IPF compared with normal samples and its number of co-expressed DE mRNAs. Red points denote up-regulated lncRNAs involved in co-expression pairs and blue points denote down-regulated lncRNAs. Cutoffs of p-value 〈 0.01 and PCC 〉 0.8 were applied to identify the co-expression pairs. The connecting line indicates the co-expression relationship between two genes, and the thickness of the line indicates the strength of the correlation coefficient. The size of the node indicates the degree of connectivity of the gene, and the higher the degree of connectivity, the larger the node. (B) Top 10 most enriched GO terms by the DE mRNAs that were co-expressed with up-regulated DE lncRNAs (above) and down-regulated DE lncRNAs (below). (C) The co-expression network between up-regulated DE lncRNAs and DE mRNAs (above), and between down-regulated DE lncRNAs and DE mRNAs (below) that were involved in the 10 GO terms. The DE lncRNAs were shown in red on the left, the co-expressed mRNAs were in the center and the mRNA-enriched GO terms were on the right. (D) Boxplots showed the expression status of 4 DE lncRNAs in IPF and normal samples.

A co-expression network analysis was used to study the involvement of lncRNAs in IPF by analyzing the relationship between differentially expressed lncRNAs and mRNAs. Genes with strong correlations may have similar functions and regulate each other in a trans manner. The criteria for the co-expression relationship were |Pearson’s correlation coefficients| > 0.8 and p-value < 0.01. Under this criteria, 527 DE lncRNAs were correlated with 1043 DE mRNAs, with 41,858 DE lncRNA-mRNA pairs, suggesting the close relationship between the two RNA types and the close relationship between DE lncRNAs and mRNAs. Compared to the down-regulated lncRNAs, the up-regulated DE lncRNAs were more highly correlated with DE mRNAs, with a correlating number of more than 120 in some cases (Fig. 2A). Some DE lncRNAs, including LINC01207, RP11–28A20.1 and LINC00939, were significantly up-regulated (-log_10_(p-value) > 2.5) and correlated with abundant DE mRNAs (> 150 DE mRNAs) (Fig. 2A). GO analysis showed that DE mRNAs co-expressed with up-regulated lncRNAs were enriched in extracellular matrix organization, tumor necrosis factor signaling, and extracellular matrix disassembly (Fig. 2B), and cytokine-cytokine receptor interaction by KEGG enrichment analysis (Supplemental Fig. 2A). DE mRNAs co-expressed with down-regulated lncRNAs were enriched in chronic inflammation response, inflammatory response regulation, TNF-mediated signaling, wound response, extracellular matrix organization, and NF-kappa B activity regulation (Fig. 2B). They were also enriched in cytokine-cytokine receptor and ECM-receptor interactions (Supplemental Fig. 2B). These signal pathways which are related to extracellular matrix and inflammation have been reported to be important pathogenesis in IPF. The up- and down-regulated lncRNAs-DE mRNAs regulatory networks were illustrated in Fig. 2C. Several DE lncRNAs were significantly changed in IPF and co-expressed with abundant DE mRNAs, including RP11–275I14.4, CTB-51J22.1, RP11–412H8.2 and XLOC_302,021 (Fig. 2D). Taken together, the result showed that the up- and down-regulated lncRNAs might function in the extracellular matrix and inflammation response-related pathways in IPF.

### LncRNA-mRNA co-expression network analysis

**Fig. 3:**
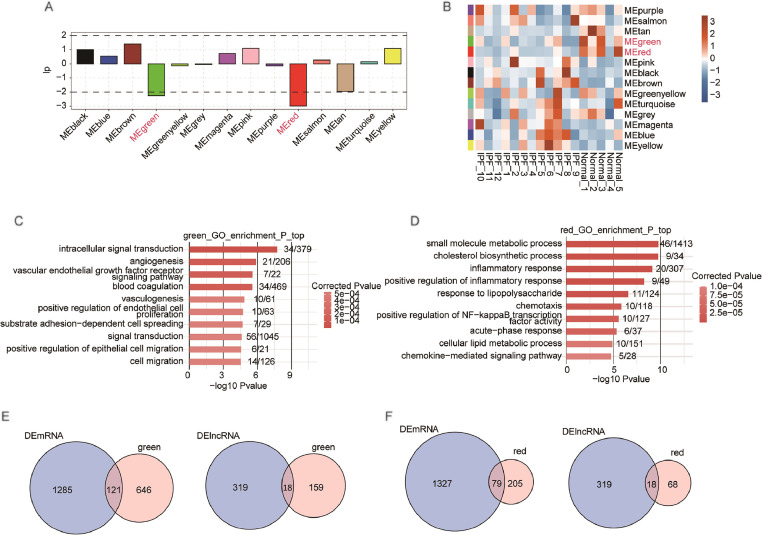
The co-expression pattern of differentially expressed genes by WGCNA. (A) Bar plot presented the significance of the correlation in the IPF samples among each pattern. Red indicates the two modules with the highest negative correlation significance. (B) The eigengene pattern of each gene co-expression module was presented by a heatmap. (C) Top 10 most enriched GO BP terms by mRNA genes from green module. (D) Top 10 most enriched GO BP terms by mRNA genes from red module. (E) Venn diagram showed the overlapped genes between DE mRNAs and mRNAs from green module (left), and DE lncRNAs and lncRNAs from green module (right). (F) Venn diagram showed the overlapped genes between DE mRNAs and mRNAs from red module (left), and DE lncRNAs and lncRNAs from red module (right).

WGCNA was utilized to analyze the co-expression of lncRNAs and mRNAs with a soft threshold of 18 (Supplemental Fig. 3A). The dendrogram of differentially expressed mRNAs was shown in Supplemental Figure 3B using hierarchical cluster analysis. Genes that co-express were color-coded. The significance of the correlation with the IPF sample phenotype among each pattern was presented in the bar plot (Fig. 3A). Among these modules, MEgreen and MEred were the two modules with the highest negative correlation significance (Fig. 3A). Eigengene patterns of MEgreen and MEred were also consistently downregulated in IPF samples (Fig. 3B). The potential functions of genes from the two modules were further explored. The MEgreen module genes showed enrichment in the VEGF receptor signaling pathway according to GO analysis (Fig. 3C). The MEred module genes were enriched in inflammation-related pathways, such as positive regulation of NF-kappa B transcription factor activity and chemokine-mediated signaling pathway (Fig. 3D). The pathways in the MEred module were related to inflammation, an important mechanism in IPF.

**Fig. 4:**
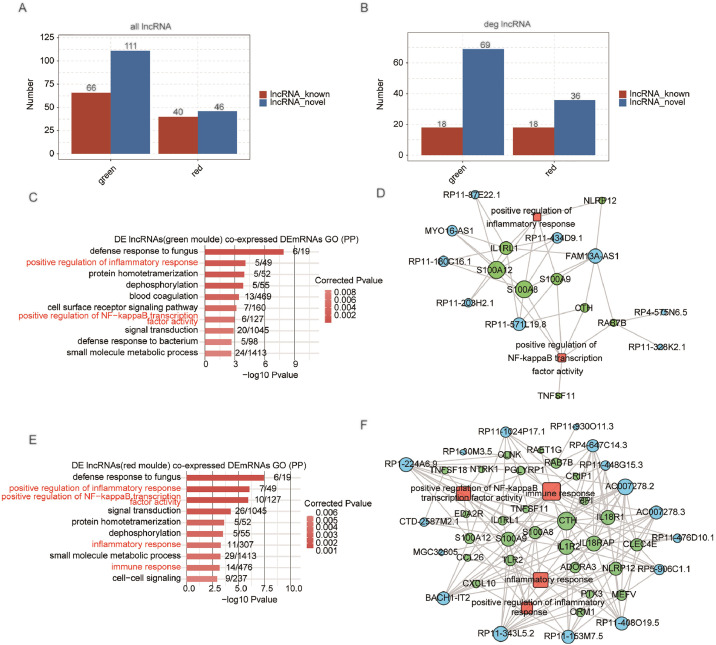
Differentially expressed lncRNAs in IPF-related modules and co-expressed DE mRNAs are enriched in immune and inflammation-related pathways. (A) Bar chart showed the number of all annotated (known) and newly predicted (novel) lncRNAs in IPF-related modules. (B) The bar chart showed the number of DE annotated (known) and newly predicted (novel) lncRNAs in IPF-related modules. (C) Top 10 enriched GO BP terms of mRNAs that were co-expressed with DE lncRNAs in the IPF-related green module. (D) The co-expression network between known DE lncRNAs (blue circle) in red modules and co-expressed DE mRNAs (green circle) in immune and inflammation-related pathways (red square). (E) Top 10 enriched GO BP terms of mRNAs co-expressed with DE lncRNAs in the IPF-related red module. (F) The co-expression network between known DE lncRNAs (blue circle) in green modules and co-expressed DE mRNAs (green circle) in immune and inflammation-related pathways (red square).

There were 121 DE mRNAs and 18 DE lncRNAs in the MEgreen module (Fig. 3E), 79 DE mRNAs and 18 DE lncRNAs in the MEred module (Fig. 3F). Of the 177 lncRNAs in the MEgreen module, 66 were annotated (known) and 111 were newly predicted (novel) (Fig. 4A). In the MEred module, 40 were known and 46 were novel (Fig. 4A). For DE lncRNAs, 18 were known and 69 were novel in the MEgreen module, and 18 were known and 36 were novel in the MEred module (Fig. 4B). Supplemental Figure 4 shows expression levels of known DE lncRNAs and mRNAs in two modules. GO terms were used to analyze potential functions of DE lncRNAs, revealing enrichment in inflammatory response and NF-kappa B transcription factor activity in the MEgreen module (Fig. 4C). Then, the hub known as co-expressed lncRNAs and mRNAs was extracted, and the regulatory network was constructed. The lncRNAs and mRNAs in the network included FAM13A-AS1, RP11–180C16.1, MYO16-AS1, and IL1RL1, S100A12, S100A8 (Fig. 4D). The co-expressed DE mRNAs in the MEred module were enriched in pathways related to inflammation, NF-κB activity, and immune response (Fig. 4E). The hub known co-expressed lncRNAs and mRNAs, including AC007278.2, BACH1-IT2, RP11–153M7.5, and CTH, TLR2, CCL26 (Fig. 4F), were also extracted. Their regulatory network was also constructed. Using WGCNA, IPF-related modules and the specific lncRNAs and mRNAs were detected, indicating that DE lncRNAs might function in inflammation-related signal pathways in IPF.

**Fig. 5:**
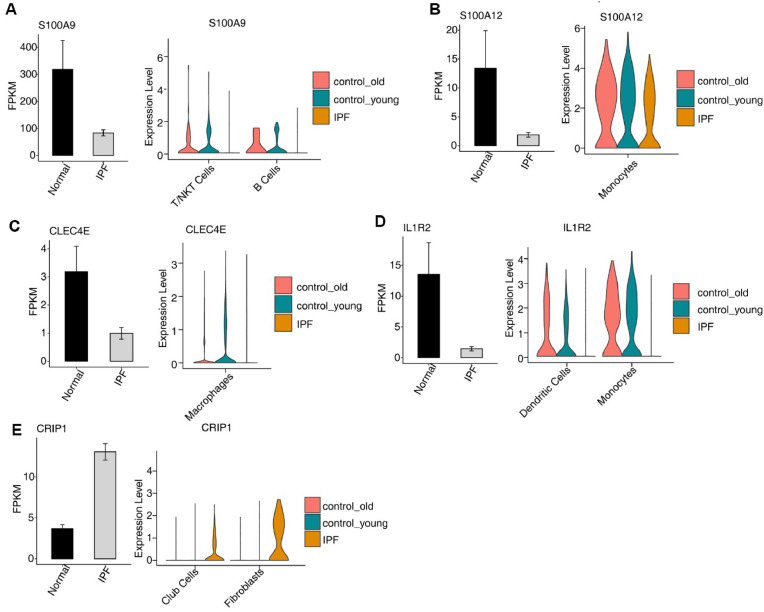
Differentially expressed DEmRNAs undergo differential changes in the specific cellular groups by scRNA-seq analysis. (A) Bar chart showed the expression of S100A9 mRNA in normal and IPF groups (left) with the decrease of S100A9 expression in T/NKT cells and B cells (right). (B) Bar chart showed the expression of S100A12 mRNA in normal and IPF groups (left) with the decrease of S100A12 expression in macrophages (right). (C) Bar chart showed the expression of CLEC4E mRNA in normal and IPF groups (left) with CLEC4E expression decreases in dendritic cells and monocytes(right). (D) Bar chart showing the expression of IL-1R2 mRNA in normal and IPF groups (left) with the decrease of IL-1R2 expression occurs in Dendritic Cells and monocytes (right). (E) Bar chart showed the expression of CRIP1 mRNA in normal and IPF groups (left) with the increase of CRIP1 expression in club cells and fibroblasts (right).

To assess the level of co-expression between different lung histiocytes of DEmRNA and DELncRNA, the authors conducted an analysis of the single-cell sequencing database. No co-expressed DElncRNAs were detected. Interestingly, the present findings indicate that the expression of DEmRNA S100A12 and S100A9 in the green module related to interstitial pulmonary fibrosis was abnormal, S100A9 expression was reduced in T/NKT cells and B-cells (Fig. 5A), while S100A12 expression was increased in monocytes (Fig. 5B). Special focus on enhancing the positive regulation of inflammation and NF-kappa B pathway activity. Furthermore, the authors found unusual levels of certain genes in the red module associated with idiopathic pulmonary fibrosis, specifically S100A12, S100A9, CLEC4E, CRIP1, and IL1R2*.* These genes are linked to inflammatory and immune responses, CLEC4E expression was reduced in macrophages (Fig. 5C), IL1R2 expression was reduced in dendritic cells and monocytes (Fig. 5D), and CRIP1 expression was elevated in rod-shaped cells and fibroblasts (Fig. 5E).

## Discussion

As the authors know, many factors, including injury or pathogen infection, increased resistance of myofibroblasts to apoptosis, and extracellular matrix deposition, contribute to the pathogenesis and development of IPF, which is enhanced by aging and genetic alterations.^30^ An in-depth examination of the expression patterns and potential regulatory roles of dysregulated lncRNAs in IPF was conducted in this study, suggesting that lncRNAs may be involved in ECM and immune/inflammatory pathways by co-expressing with related genes and, consequently, the dysregulation of lncRNAs can be closely related to IPF development. Importantly, the results of PCA showed that DE lncRNAs can be well separated in the IPF and control specimens, thus suggesting that lncRNAs could also be potentially diagnostic biomarkers in IPF.

The present hypothesis that lncRNAs contribute to the pathology in IPF is well supported by the previous multiple studies. There is increasing evidence of lncRNAs' role in pulmonary fibrosis, such as lnc949,^31^ ZEB1-AS1,^11^ and AP003419.16.^12^ Interestingly, one previous study reported that 272 lncRNAs, including the 42 newly identified lncRNAs, were differentially expressed in bleomycin-induced pulmonary fibrosis and 59 lncRNAs and 131 mRNAs were co-expressed in the lung fibrosis.^9^ In the present study, the authors identified 541 differentially expressed lncRNAs in IPF, including 201 up-regulated and 340 down-regulated lncRNAs. These results may provide new understanding of how lncRNAs contribute to pulmonary fibrosis development and treatment.

Recent findings indicate that lncRNAs can influence gene expression through either nearby (cis-acting) or distant (trans-acting) mechanisms.^32^, ^33^, ^34^ IPF-associated mRNAs and lncRNAs were predicted by analyzing co-expression networks. Finally, 527 DE lncRNAs were identified and co-expressed with 1043 DE mRNAs using their expression values from three groups. Co-expressed DE mRNAs of up-regulated DE lncRNAs indicated that they were enriched in pathways associated with extracellular matrix remodeling, which is related to the pathogenetic involvement in pulmonary fibrosis.^35^ DE mRNAs that co-expressed with down-regulated lncRNAs can primarily initiate multiple inflammatory responses in this study, including chronic inflammation response, positive regulation of chronic inflammation response, and NF-κB transcription factor activity, which are among the factors affected by chronic inflammation.

Additionally, four potential lncRNAs were newly identified with significant expression changes in IPF, including RP11–275I14.4, CTB-51J22.1, RP11–412H8.2 and XLOC_302,021. Noticeably, these lncRNAs were co-expressed with abundant DE mRNAs. Their expression levels and identified functions are associated with a range of diseases (e.g., cancer), albeit the DE lncRNAs involved in pulmonary fibrosis have not previously been reported. It is known that RP11–275,114.4 and CTB-51J22.1 are highly expressed in endometrial carcinoma, and both lncRNAs may play a role in cancer progression.^36^ However, no previous studies mentioned the important functions of RP11–412H8.2 and XLOC_302,021, which were identified in the present study, indicating their potential functions in IPF. The aforementioned lncRNAs have such regulatory roles in cancers and other diseases, as well as two lncRNAs with unknown function but significantly dysregulated in IPF, implying they may have similar roles in pulmonary fibrosis development and progression.

DE lncRNAs are linked to DE mRNAs through co-expression analysis, aiding in the prediction of lncRNA functions.^37^ In this study, WGCNA was used to study the relationship between lncRNA and mRNA in IPF.^27^ IPF-related modules were identified, with DE mRNAs in the MEgreen module showing enrichment in inflammatory response and NF-κB pathways. The pathways in the MEred module were related to the inflammation response, an important mechanism involved in IPF. IPF-related modules and specific lncRNAs and mRNAs were detected by WGCNA, indicating that DE lncRNAs might function in inflammation-related signal pathways in IPF. Therefore, these findings suggest that certain lncRNAs in the co-expression network play a role in pulmonary fibrosis by influencing extracellular matrix remodeling and inflammation. The hub of co-expressed lncRNAs and mRNAs was identified, and a regulatory network was created. The lncRNAs and mRNAs in the network included FAM13A-AS1, MYO16-AS1, IL1RL1, S100A12, S100A8, AC007278.2 and RP11–153M7.5, respectively. FAM13A-AS1 is a novel biomarker for the prognosis of thyroid cancer,^38^ and MYO16*-*AS1 acts as an oncogenic lncRNA in bladder cancer,^39^ AC007278*.2* and RP11–153M7.5 are associated with SLE.^40^^,^^41^ Through network analysis, the hub lncRNAs may regulate the expression of co-expressed mRNAs through multiple mechanisms, such as acting as a sponge of miRNAs, modulating mRNA by transcriptional regulation, or chromatin modification.^32^

At the conclusion of this study, the authors observed aberrant expression of DEmRNAs in the red and green modules associated with IPF through the analysis of the scRNA-seq dataset. Notably, genes such as S100A12, S100A9, CLEC4E, CRIP1, and IL1R2 exhibited abnormal expression patterns. GO analysis revealed enrichment of genes in inflammatory and immune response processes, as well as positive regulation of inflammatory factor activity and NF-κB pathways. Furthermore, the present findings demonstrated that CLEC4E expression was reduced in macrophages, IL1R2 expression was reduced in dendritic cells and monocytes, and CRIP1 expression was increased in club cells and fibroblasts. S100A9 expression was decreased in T/NKT cells and B-cells, whereas the expression of S100A12 was observed to be increased in monocytes. These observed changes in signaling pathways align with the physiological functions associated with immune cells expressing these mRNA molecules. Previous research has demonstrated the significant involvement of S100A8/A9 in initiating inflammation and facilitating the development of a self-perpetuating inflammatory cycle that contributes to fibrosis, primarily through the activation of neutrophils and macrophages.^42^ Additionally, S100A12 exhibited predominant and elevated expression levels in monocytes. There have been limited prior investigations conducted on the other three genes. Notably, the co-expression of DElncRNAs with these five DEmRNAs (e.g., RP11–434D9.1, RP11–203H2.1, MYO16-AS1, RP11–180C16.1, RP11–87E22.1, RP11–571L19.8, FAM13A-AS1, etc.) was not observed, indicating that these DEmRNAs may be regulated by other factors or indirectly by lncRNAs. Thus, identifying DElncRNAs that regulate mRNA targets could provide new research opportunities for diagnosing and treating IPF.

Existing studies have reported divergent roles of certain lncRNAs in IPF compared to these findings. For instance, some research suggests that specific lncRNAs primarily influence IPF through the modulation of cell proliferation-related pathways. LncRNA NONMMUT060091 (PFI) was found to be significantly downregulated in mouse models of lung fibrosis, while it was upregulated in human lung fibroblasts. This lncRNA plays a critical role in the pathogenesis of pulmonary fibrosis by interacting with and modulating the expression of Serine/arginine-Rich Splicing Factor-1 (SRSF1), a protein primarily involved in RNA splicing processes.^43^ LncRNA DACH1 is significantly down-regulated in the lungs of patients with IPF as well as in experimental mouse models of pulmonary fibrosis. Overexpression of lncRNA DACH1 has been shown to attenuate the aberrant activation, collagen deposition, and differentiation of mouse lung fibroblasts induced by TGFβ1.^44^ This discrepancy may be attributed to several factors, including variations and bias in sample sources. The samples utilized in different studies may differ in terms of disease stages and patient-specific characteristics, such as age, comorbidities, and treatment history. More samples should be considered in future studies.

The present study identified dysregulated lncRNAs and their potential targets in pulmonary fibrosis, expanding the knowledge of their functional properties. However, the limited sample size and undetermined clinical characteristics of patients such as age, smoking status, and treatment history, of the RNA-seq dataset were shortcomings in this study to identify the highly confident DE lncRNAs in IPF, and more samples are needed for validation in the future. Another limitation of the study is the necessity of further experiments, such as expression validation or knockdown cellular or animal models, to validate the functions of differentially co-expressed lncRNA-mRNA pairs. These pairs have validated functions in other diseases, indicating their potential relevance in IPF progression. Further research is required to validate the interaction between these DE lncRNAs and mRNAs at various levels using larger and ethnically diverse cohorts. Meanwhile, predictive modeling or biomarker panels should be constructed on these DE lncRNAs to validate their potential in IPF diagnosis. The study results offer insights for studying the molecular mechanisms of pulmonary fibrosis and suggest new directions for clinical research on potential therapeutic targets.

## Conclusion

This study analyzed the genome-wide profiling of lncRNAs in IPF, detecting abundant lncRNAs and building co-expression networks with DE mRNAs. The study found that DE lncRNAs and their co-expressed DE mRNAs were enriched in pathways related to inflammation, providing insight into the pathogenetic mechanisms of pulmonary fibrosis and, importantly, this study offers new avenues for investigating the development of IPF with lncRNAs in future preclinical and clinical studies.

## Declarations

Ethics approval and consent to participate: The public data sets used in this study have passed the original research ethics review and all data have been anonymized in compliance with the Helsinki Declaration.

## Informed consent and patient details

Not applicable.

## Consent for publication

Not applicable.

**Availability of data and materials:** The datasets used and/or analyzed during the current study are available from the corresponding author on reasonable request.

## Funding

Henan Province Medical Science and Technology Key Project (Jointly Constructed by the Province and the Ministry) (SBGJ202402013).

## Conflicts of interest

The authors declare no conflicts of interest.

## Appendix Supplementary materials

Image, application 1
