# Supplementary material for: Potential function exploration of lncRNAs in idiopathic pulmonary fibrosis: insights from whole transcriptome sequencing data analysis
Source: Clinics (Sao Paulo). 2025 Aug 8;80:100732. doi: 10.1016/j.clinsp.2025.100732 (PMC12356403; doi:10.1016/j.clinsp.2025.100732)

**CLINICS-D-24-00315_Supplementary Material**

**Supplemental Figure 1 Functional pathways of differentially expressed genes in IPF and normal samples.** (A) The heatmap showed the Pearson’s correlation coefficients among IPF and normal samples. (B‒C) The GO analysis of DE mRNAs was divided into up- (B) and down-regulated (C) genes. (D) The bar chart showed the number of different DE lncRNAs from up- and down-regulated genes, including the known lncRNAs and novel lncRNAs).


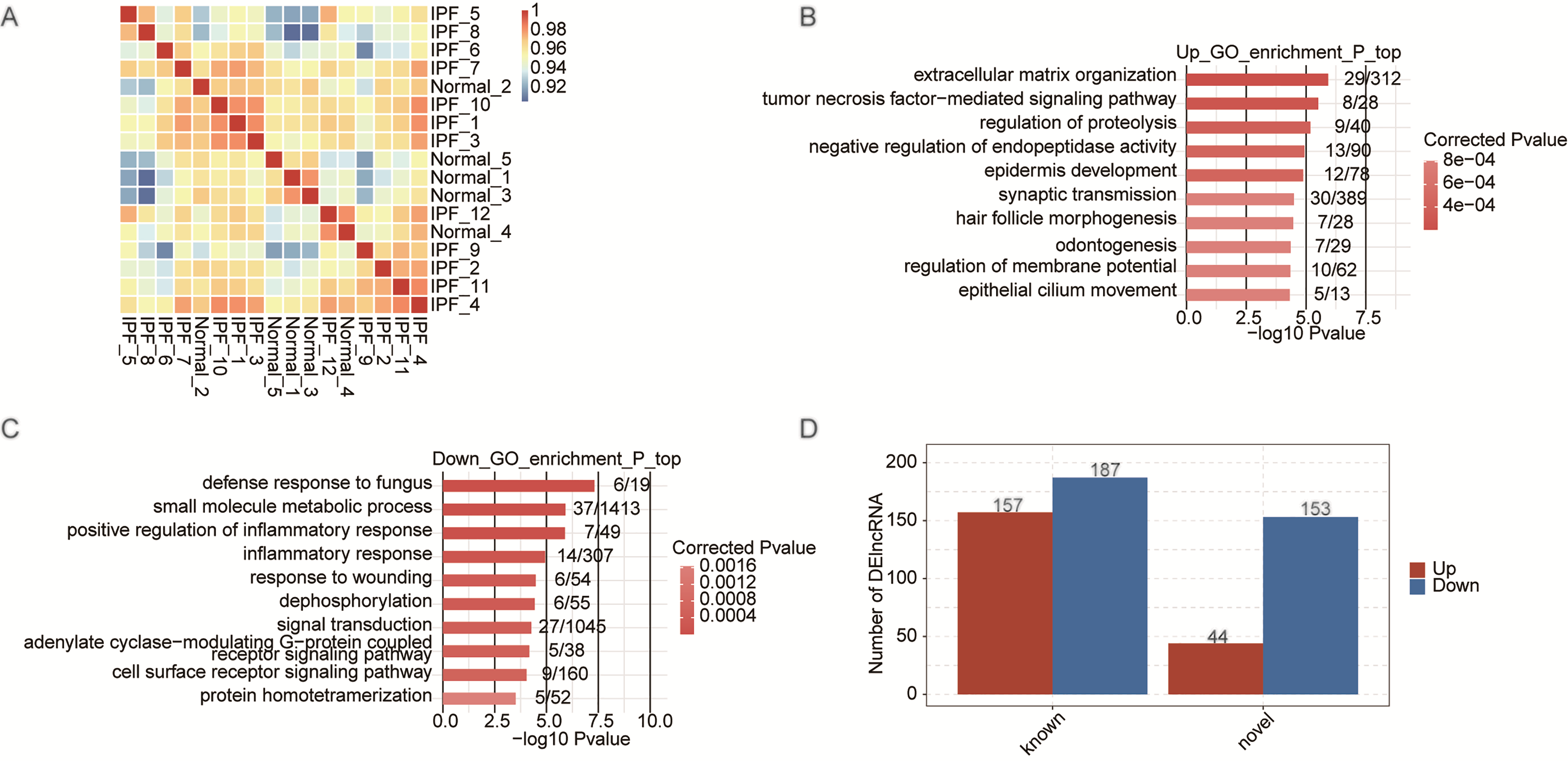


**Supplemental Figure 2** **Top 10 most enriched KEGG pathways by the DE mRNAs that were co-expressed with DE lncRNAs.** (A) Top 10 most enriched KEGG pathways by the DE mRNAs that were co-expressed with up-regulated DE lncRNAs. (B) Top 10 most enriched KEGG pathways by the DE mRNAs that were co-expressed with down-regulated DE lncRNAs.


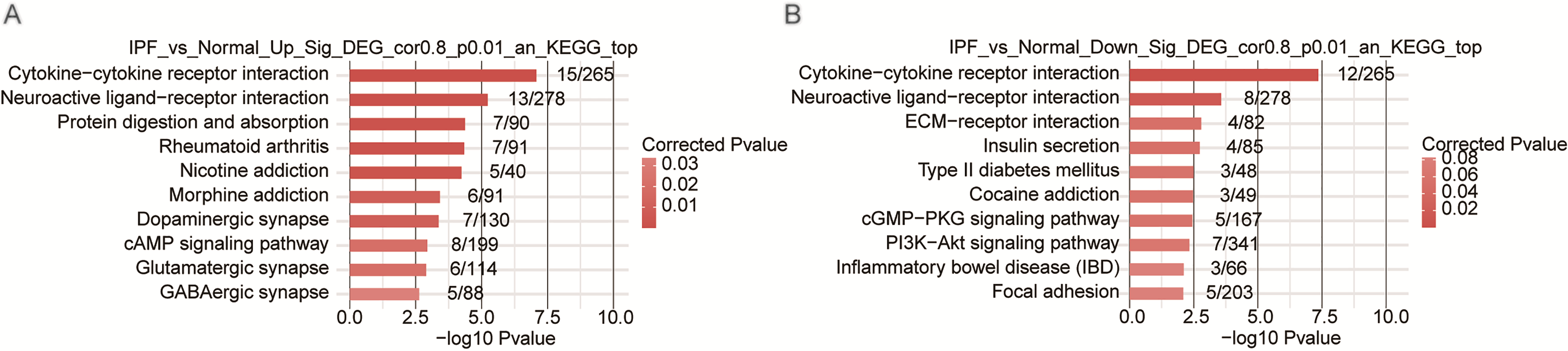


**Supplemental Figure 3 The co-expression pattern of differentially expressed genes by WGCNA.** (A) Scale-free topology model fit analysis showed the soft threshold (power) for lncRNAs and mRNAs based on scale-free topology criteria. (B) Dendrogram of all differentially expressed mRNAs by hierarchical cluster analysis. Each co-expressed gene was assigned to a module color.


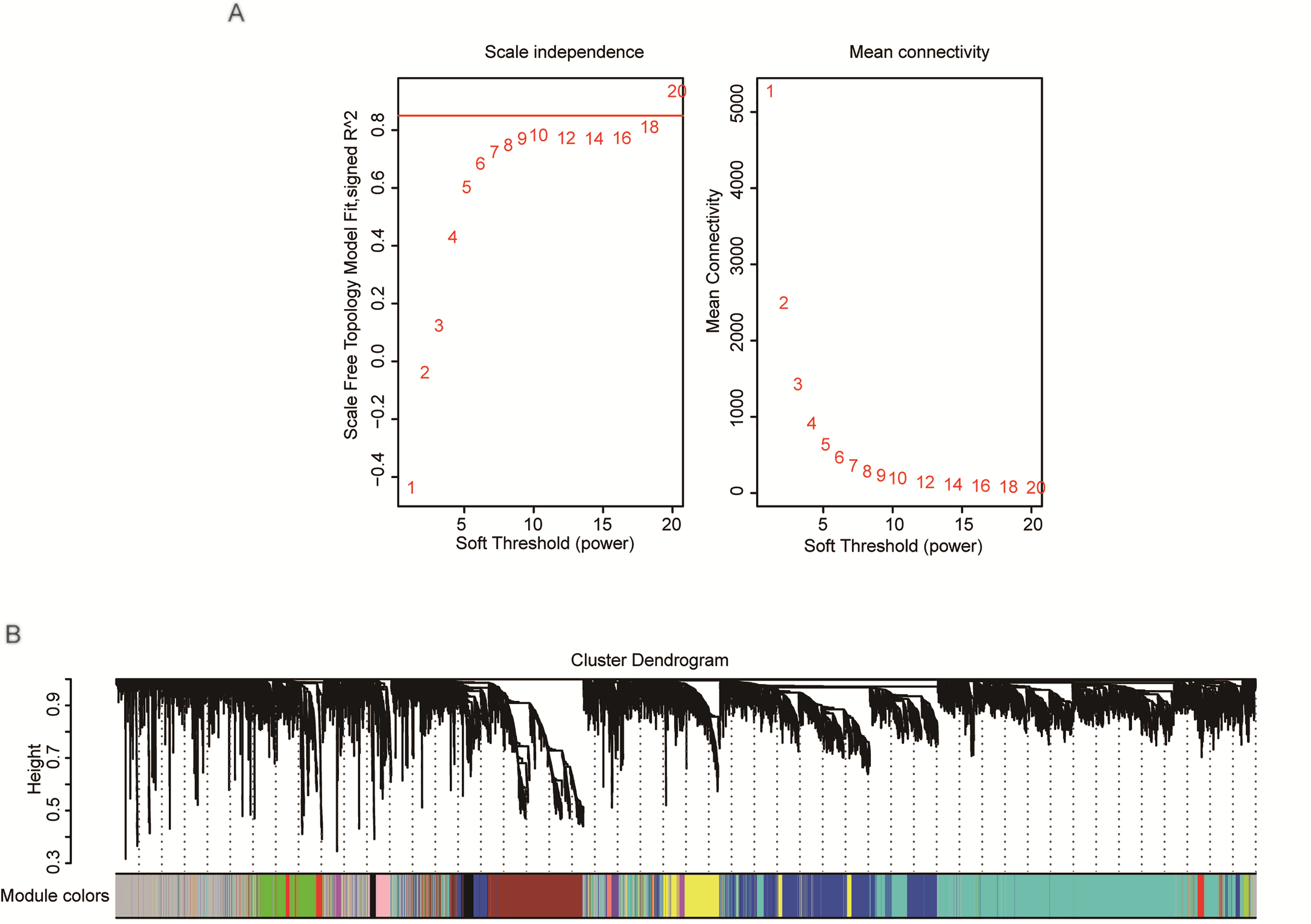


**Supplemental Figure 4** **Expression heatmaps of DE lncRNAs and DE mRNAs in IPF and normal samples.** (A) Heatmap showed the expression status of 9 DE lncRNAs from green model in IPF and normal samples. (B) Heatmap showed the expression status of 8 DE mRNAs from green model in IPF and normal samples. (C) Heatmap showing expression status of 16 DE lncRNAs from red model in IPF and normal samples. (D) Heatmap showing expression status of 27 DE mRNAs from red model in IPF and normal samples.


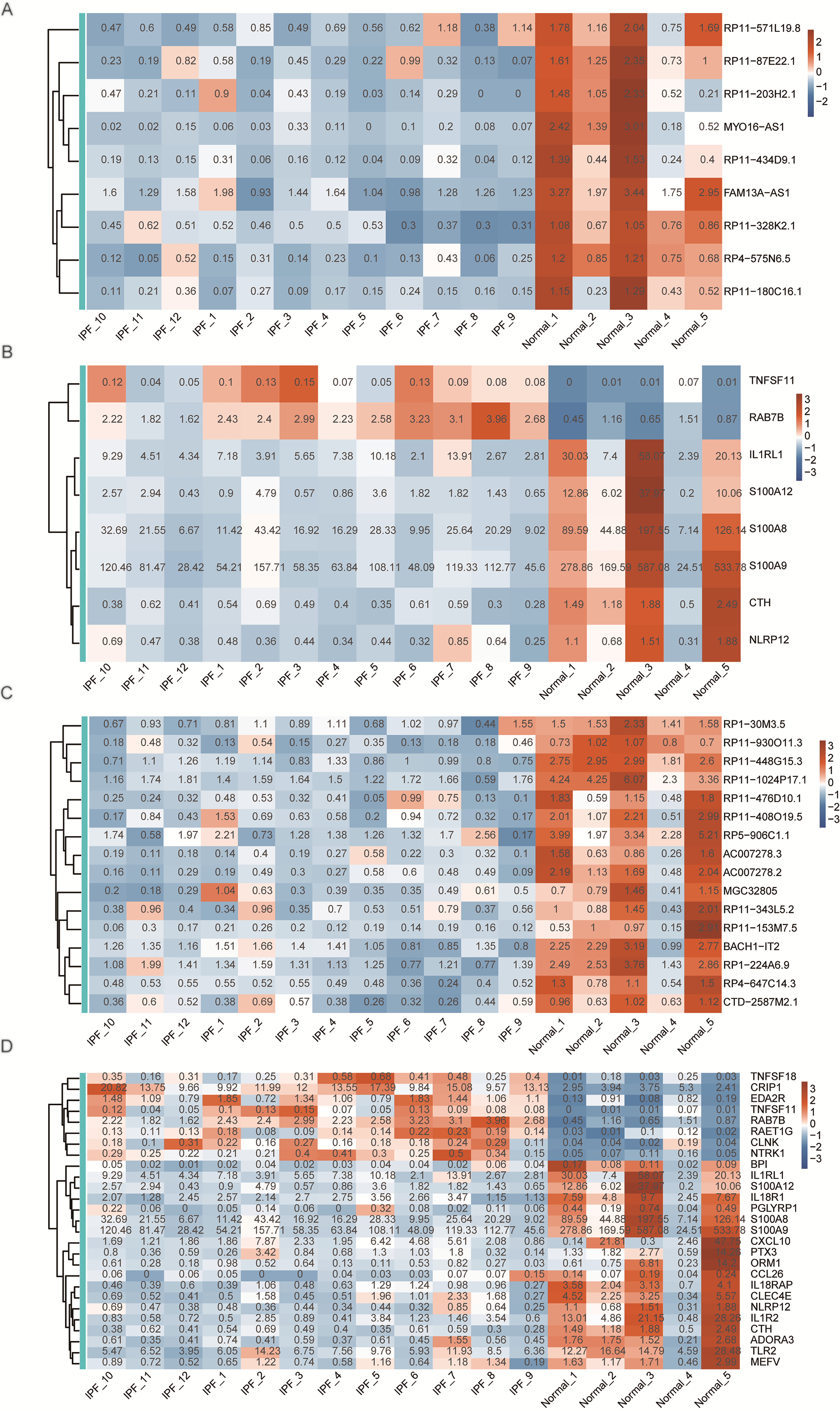

Supplement: Supplementary file 1 [file mmc1.doc]
